# Osteoporosis Among Bahraini Women Based on Bone Mineral Density Measurements: A Retrospective Study

**DOI:** 10.7759/cureus.31368

**Published:** 2022-11-11

**Authors:** Tareq Al Taei, Omran Sarwani, Hamed Almalki, Mohamed Alameer, Naeema Ali, Najla Alomani, Zahra Alyusuf, Wafa Hasan, Reem Maki, Sarah Al Mail

**Affiliations:** 1 Radiology, Salmaniya Medical Complex, Busaiteen, BHR; 2 Radiology, Salmaniya Medical Complex, Manama, BHR; 3 Radiology, Salmaniya Medical Complex, Riyadh, SAU

**Keywords:** world health organization, bone mineral density (bmd), dual-energy x-ray absorptiometry (dexa), osteopenia, osteoporosis

## Abstract

Objectives

The objective is to estimate the prevalence of osteoporosis among women in Bahrain who are aged ≥18 years.

Methods

In this retrospective study conducted at Salmaniya Medical Complex, a total of 590 Bahraini women were enrolled. Their bone mineral density measurements were obtained through dual-energy x-ray absorptiometry (DEXA) performed between January 2017 and December 2017. Six sites were chosen as the measurement targets. Patients were diagnosed with osteoporosis if their T-score was > -2.5 according to the World Health Organization guidelines.

Results

Osteoporosis was diagnosed in 27.1% of the patients; 53.2% had osteopenia, 0.3% had severe osteoporosis, and 19.3% had normal bone conditions. The prevalence of osteopenia as well as osteoporosis increased with age.

Conclusion

Osteoporosis and osteopenia are common among Bahraini women. This study provides useful information on the prevalence of osteoporosis among Bahraini women. Major steps by health authorities in the country are needed to reduce morbidity and improve the quality of life.

## Introduction

Osteoporosis is a common bone disease affecting approximately 200 million people worldwide [1]. The World Health Organization defines osteoporosis as bone mineral density (BMD) lower than the average (from a study of mainly Caucasian women) by ≥2.5 standard deviations and could be considered an evolution of osteopenia [2]. Osteoporosis causes decreased BMD and fragility of bones, thus increasing the incidence of fractures due to microarchitectural deterioration.

Osteoporosis is one of the most common diseases affecting bones, affecting millions worldwide and having a detrimental effect on patients’ well-being and lifestyle [3]. Since osteoporosis is a systemic disease, a multidisciplinary approach is required to diagnose and treat it. Multiple specialties, including internal medicine, endocrinology, gynecology, orthopedic surgery, and radiology, need to be involved. Different treatment approaches for osteoporosis have been a major topic of discussion since the early 1970s [4].

Osteoporosis is considered a chronic disease in which bone loss occurs silently and relentlessly until a fracture occurs; therefore, appropriate management and measures to prevent it are of utmost importance. Osteoporosis is classified into two main types: primary or idiopathic osteoporosis, the most common type, and secondary osteoporosis, which may be caused by several diseases [5]. As the population ages, the prevalence of chronic diseases increases as well. The 2010 “Global Burden of Disease Study” reported that the burden of noncommunicable diseases in the Arab world has increased and is estimated to increase further in the coming years [6].

Osteoporosis and its subsequent consequences are a burden on medical systems worldwide. In Europe alone, osteoporosis accounts for 1.5% of the medical burden, with an estimated two million disability-adjusted life years lost per year [7,8]. In the USA, approximately 1.5 million fractures occur annually among patients with osteoporosis, with an even higher incidence in pregnant women [9]. Estimations from 2000 showed that approximately nine million cases of osteoporotic fractures occur worldwide annually, with projections that the number of cases will double by 2040 [10].

Previous studies have shown that the prevalence of osteoporosis varies widely among ethnic and geographic groups. Among the Arab Gulf countries, a report from Saudi Arabia reported an osteoporosis incidence of 33%, whereas a study from Kuwait found an incidence of 20.2% [11,12]. Another study examining the prevalence in a younger population of the United Arab Emirates (UAE) revealed that 24% of the participants had osteopenia and approximately 2% had osteoporosis [13]. The prevalence in first-world countries such as the United States of America (USA) is 16%, however, it is 38% in Japan [14].

In the kingdom of Bahrain, the prevalence data for osteoporosis are limited, and only a few studies were conducted that showed different results [15-17]. By conducting this study, we aimed to accurately evaluate the prevalence of osteoporosis in Bahrain and help the health authorities implement plans to reduce the patients’ morbidity and improve their quality of life.

## Materials and methods

Dual-energy x-ray absorptiometry (DEXA) scans were retrospectively reviewed over a one-year period between January 2017 and December 2017 at Salmaniya Medical Complex, Kingdom of Bahrain. The participants’ data, including age, gender, images, and reports, were retrieved from the picture archiving and communication system after obtaining approval from the Institutional Review Board. Bahraini women aged ≥18 years were included in the study. Patients aged < 18 years and men were excluded. All the patients were scanned using a GE machine (General Electric Lunar, Chicago, IL, USA), and the GE NHANES III software was used for the diagnosis. The DEXA scans were interpreted in terms of the T-score. According to the World Health Organization guidelines (Table 1), osteoporosis was diagnosed if the T-score was > -2.5. Six sites were chosen as targets for measurement: the lumbar spine, left and right upper femur, left distal radius and left and right femur neck. The scans were reviewed by a single general consultant radiologist with >25 years of experience.

**Table 1:** World Health Organization data on the interpretation of T-scores obtained from dual-energy x-ray absorptiometry (DEXA) scans

Diagnosis	T-score
Normal	> - 1.0
Osteopenia	< - 1.0 > - 2.5
Osteoporosis	< - 2.5
Severe osteoporosis	< - 2.5 plus fragility fracture

Descriptive data analyses were performed using IBM SPSS V.28, in which demographic and radiologic parameters were explored. Categorical variables are presented as frequency and percentage distributions for each category. ANOVA test was used for the analysis of variance results.

Continuous variables, such as BMD and T-score for various bones, are presented as central measure tendencies and measures of dispersions (mean, standard deviation). The statistical significance of the extent of the difference between the mean scores of different subgroups was assessed using t-test of independent samples. The level of statistical significance was set at an alpha value of 0.05.

## Results

A total of 640 patients underwent DEXA between January and December 2017, of which 590 patients with a median age of 57.2 ± 12.4 years were enrolled in this study. Based on age, patients were divided into four subgroups (<20 years, 21-40 years, 41-60 years, and >60 years), with those aged ≥41 years constituting 89.2% of the entire sample (Table 2). Approximately 27.1% had osteoporosis, 53.2% had osteopenia, 0.3% had severe osteoporosis, and 19.3% had normal bone conditions (Table 3). Among patients aged >60 years, the prevalence of osteoporosis was twice that of their 41- to 60-year-old counterparts (41.1% vs. 19.7%, p = 0.004). Similarly, the prevalence of normal bone conditions decreased significantly as age progressed.

**Table 2:** Distribution of patients based on age

Age group	Frequency	Percentage
0–20 years	7	1.2%
21–40 years	59	9.7%
41–60 years	310	51.1%
>60 years	231	38.1%

**Table 3:** Results based on age groups

Variables	Normal	Osteopenia	Osteoporosis	Severe osteoporosis
	114 (19.3%)	314 (53.2%)	160 (27.1%)	2 (0.3%)
p-value	0.532	0.360	0.950	-
Age group				
0–20 years	1 (14.3%)	4 (57.1%)	2 (28.6%)	0 (0%)
21–40 years	21 (35.6%)	28 (47.5%)	10 (16.9%)	0 (0%)
41–60 years	73 (23.5%)	175 (56.5%)	61 (19.7%)	1 (0.3%)
>60 years	120 (10.8%)	317 (47.6%)	168 (41.1%)	2 (0.4%)

Lumbar spine (31.9%), followed by the left femoral neck (15.6%), showed the highestThe lumbar prevalence of osteoporosis. The maximum prevalence of osteopenia was noted at the left femoral neck (53.7%), followed by the right femoral neck (50.9%) (Table 4).

**Table 4:** Results based on the location of the scan

	Normal	Osteopenia	Osteoporosis
Lumbar spine	26.8%	41.3%	31.9%
Left femoral neck	30.7%	53.7%	15.6%
Right femoral neck	35.7%	50.9%	13.4%
Left distal radius	37.7%	49.8	12.5%

The four groups differed significantly in terms of their mean BMD and T-scores across the six anatomical locations according to the analysis of variance (ANOVA) results. For example, at the lumbar spine, the normal group showed a significantly higher mean BMD (1.18 ± 0.17) than the osteopenia (1.01 ± 0.11), osteoporosis (0.81 ± 0.14), and severe osteoporosis (0.47 ± 0.05) groups. The BMD decreased significantly moving from one group to the next, and the T-score significantly increased in the same direction for all six anatomic locations (Table 5).

**Table 5:** Distribution of bone mineral density (BMD) and T-scores *: Intended to flag levels of significance for three of the most commonly used levels. If a p-value is less than 0.001, it is flagged with three stars (***).

		Normal		Osteopenia		Osteoporosis		Severe osteoporosis		
		Mean	SD	Mean	SD	Mean	SD	Mean	SD	p-value
L-spine BMD	1.18		± 0.17	1.01	± 0.11	0.81	± 0.14	0.47	± 0.05	< 0.001***
T-score	0.11		± 0.85	-1.35	± 0.82	-3.04	± 1.17	-5.90	± 0.42	< 0.001***
Left femur BMD	1.09		± 0.10	0.92	± 0.09	0.76	± 0.11	0.36	± 0.07	< 0.001***
T-score	0.54		± 0.84	-0.64	± 0.84	-1.97	± 0.94	-5.15	± 0.49	< 0.001***
Left femur neck BMD	1.01		± 0.10	0.84	± 0.16	0.71	± 0.11	0.40	± 0.04	< 0.001***
T-score	-0.15		± 0.62	-1.34	± 0.60	-2.26	± 0.94	-4.60	± 0.28	< 0.001***
Right femur BMD	1.09		± 0.10	0.92	± 0.10	0.75	± 0.11	0.36	± 0.07	< 0.001***
T-score	0.57		± 0.82	-0.72	± 0.84	-1.96	± 0.99	-5.20	± 0.57	< 0.001***
Right femur neck BMD	1.02		± 0.14	0.85	± 0.10	0.70	± 0.19	0.41	± 0.00	< 0.001***
T-score	-0.02		± 0.60	-1.33	± 0.69	-2.32	± 0.77	-4.50	± 0.00	< 0.001***
Left radius BMD	0.69		± 0.08	0.60	± 0.09	0.52	± 0.12	0.27	± 0.23	< 0.001***
T-score	-0.13		± 1.12	-1.42	± 1.26	-2.93	± 1.67	-6.85	± 3.75	< 0.001***

## Discussion

This study concluded that a considerable proportion (27%) of the patient had osteoporosis. In the three previous studies on this topic conducted in Bahrain, sample size differences could explain the varying results obtained. The first of these studies was conducted in 2009 on 17 postmenopausal women [15]. The second study was a retrospective analysis of 205 patients, which showed a higher prevalence of osteoporosis than that found in our study [16]. The final study showed a significantly lower prevalence of osteoporosis in approximately 892 women [17].

Compared with international data, the date of the present study indicates a higher prevalence of osteoporosis than in the USA (10.3%); however, a lower prevalence than that in China (34.6%) and Japan (38%) was noted [14]. A similar study conducted in the UAE among a population with a mean age of 42 years reported that the prevalence of osteopenia ranged between 22% and 24%, whereas that of osteoporosis ranged between 2.5% and 3% [13]. These numbers are lower than those reported in the present study.

Another study published in the Kingdom of Saudi Arabia on participants aged ≥50 years reported a slightly higher prevalence (34%) of osteoporosis and a lower prevalence of osteopenia (36.6%) than that reported in our study [14,18,19]. In a recent study conducted in Jordan, the prevalence of osteoporosis among Jordanian women was the lowest prevalence among others in Middle Eastern countries and was reported to be 13.5% [20].

It is important to note that when comparing prevalence rates, the varying methods used in estimating BMD must be considered since they can contribute to differences in the results obtained. For instance, some studies and institutes calculate BMD from the femur, whereas others calculate BMD from the spine, and some use either ultrasonography or DEXA for calculation [4].

A physical examination coupled with a comprehensive history focusing on obtaining information regarding secondary risk factors which are potential causes for secondary bone loss from the patient. A thorough social history should also be obtained with attention to smoking history and chronic alcohol intake. The family history may reveal a hereditary bone disorder or a predilection of the family towards processes such as osteoporosis. The patient should be asked about any prior diagnosed fractures but also with a focus given on low-energy ground-level fall mechanisms and any fractures which occurred after 40 years of age. Physical examination, although routinely done, rarely reveals any changes until the disease has advanced [21,22].

In healthy individuals without risk factors, experts recommend starting the screening process for women at the age of 65 years of age and men at the age of 70. It should be noted that the United States Preventative Services Task Force did not find sufficient evidence for them to recommend screening men of any age group. Patients with evident risk factors or a high score on an osteoporosis risk assessment test should commence a screening program earlier [23].

Lifestyle changes are very important to recommend, and help encourage, in all patients. Weight-bearing physical activity and exercise that improves balance, such as yoga and tai-chi, have been shown to be beneficial. Treatment, including certain medications, can be offered to help with both smoking and alcohol cessation if the patients meet the criteria. Recommend calcium and Vitamin D3 to all patients, and patients who are vitamin D deficient should have a goal of raising their levels back to normal [22]. Although our study is limited by its retrospective nature and the population being selected from a single center, using this method has helped provide an accurate and definitive prevalence rate of osteoporosis among Bahraini women.

## Conclusions

Osteopenia and osteoporosis are common among Bahraini women. This study provides useful information on the prevalence of osteoporosis among Bahraini women. In the future, we hope that health authorities in the country will conduct further studies and take major steps toward the early diagnosis and treatment of this condition, thus limiting its morbidity and effects on quality of life, especially in the elderly population.
